# Complete genome sequences of gastric non-*Helicobacter pylori Helicobacter* species, *H. suis* HS1^T^, *H. heilmannii* ASB1^T^, and *H. ailurogastricus* ASB7^T^

**DOI:** 10.1128/MRA.00938-22

**Published:** 2025-05-20

**Authors:** Sae Aoki, Emiko Rimbara, Hidenori Matsui, Tsuyoshi Kenri, Masato Suzuki

**Affiliations:** 1 Department of Bacteriology II, National Institute of Infectious Diseases, Japan Institute for Health Security, Musashimurayama, Tokyo, Japan; 2 Antimicrobial Resistance Research Center, National Institute of Infectious Diseases, Japan Institute for Health Security, Higashimurayama, Tokyo, Japan; Indiana University Bloomington, Bloomington, Indiana, USA

**Keywords:** *Helicobacter*, NHPH, gastric diseases, humans, animals

## Abstract

Non-*Helicobacter pylori Helicobacter* species (NHPH) are known to be present in the stomachs of humans and animals and are associated with their health. Here, we report complete genome sequences of type strains of *Helicobacter suis*, *Helicobacter heilmannii*, and *Helicobacter ailurogastricus*, which are major gastric NHPH species associated with human gastric diseases.

## ANNOUNCEMENT

Non-*Helicobacter pylori Helicobacter* (NHPH) species are known to be present in the stomachs of humans and animals and are associated with their health (1–4). Among NHPH species, *Helicobacter suis*, *Helicobacter heilmannii*, and *Helicobacter ailurogastricus* are the most prevalent species infecting the human stomachs and causing gastric diseases, including peptic ulcers, chronic gastritis, gastric cancers, and gastric mucosa-associated lymphoid tissue (MALT) lymphoma (2–4). Draft genome sequences of type strains of those above-mentioned gastric NHPH species have been available (5), but their complete genome sequences are not so far. Here, we report the complete genome sequences of type strains of *H. suis*, *H. heilmannii*, and *H. ailurogastricus* for the first time.

*H. suis* HS1^T^ (=DSM 19735^T^), *H. heilmannii* ASB1^T^ (=DSM 24751^T^), and *H. ailurogastricus* ASB7^T^ (=DSM 100489^T^) were originally isolated from a pig, a cat, and a cat, respectively, in Belgium. These strains obtained from the Leibniz Institute DSMZ–German Collection of Microorganisms and Cell Cultures in Germany were grown in Brucella broth medium containing 20% fetal bovine serum (Gibco), Skirrow supplement (Oxoid), Vitox supplement (Oxoid), and 0.05% HCl (FUJIFILM Wako; final pH=∼5.0). Genomic DNAs were extracted using Genomic-tips and Genomic DNA Buffer Set (QIAGEN). Whole-genome sequencing was performed using MiniSeq (Illumina) with MiniSeq High Output Reagent Kit (300-cycles) and MinION Mk1B (Oxford Nanopore Technologies) with the R9.4.1 flow cell. The libraries for Illumina (150 bp paired-end, insert size of 500 to 900 bp) and ONT sequencing were prepared using the Nextera XT DNA Library Prep Kit and the Rapid Barcoding Kit (SQK-RBK004), respectively.

Illumina reads (1,933,020, 237,132, and 412,930 reads for HS1^T^, ASB1^T^, and ASB7^T^) were trimmed using Trimmomatic v0.39 (https://github.com/usadellab/Trimmomatic). ONT reads (56,198, 31,721, and 37,240 reads; N50 = 10,473.0, 20,074.0, and 13,015.0 bp for HS1^T^, ASB1^T^, and ASB7^T^) were basecalled using Guppy v4.2.2, were trimmed using Filtlong v0.2.1 (https://github.com/rrwick/Filtlong) with parameters “--min_length 1000 --min_mean_q 10”, and were assembled *de novo* using Canu v1.8 (https://github.com/marbl/canu). The overlap regions in the assembled contigs were detected using LAST v1407 (https://gitlab.com/mcfrith/last) and then trimmed manually, resulting in circularized genomes. Sequencing errors were corrected by Racon v1.4.20 (https://github.com/isovic/racon) twice using ONT reads and then corrected by Pilon v1.20.1 (https://github.com/broadinstitute/pilon) twice using Illumina reads, resulting in their complete genome sequences. Default parameters were used except where otherwise noted.

The quality of the resulting complete chromosome sequences of *H. suis* HS1^T^ (accession no. AP026769), *H. heilmannii* ASB1^T^ (accession no. AP026684), and *H. ailurogastricus* ASB7^T^ (accession no. AP026687) were assessed by CheckM v1.1.3 (https://github.com/Ecogenomics/CheckM), and genome completeness and contamination were estimated at 98.5% and 0.18%, 98.48% and 0%, and 98.46% and 0.36%, respectively (Table 1). Their complete genome sequences consisted of one chromosome and two plasmids, one chromosome and two plasmids, one chromosome and three plasmids (average sequencing depth of ONT sequencing: 40×, 95×, and 103×) with total sequences of 1,689,191, 1,660,951, and 1,609,065 bp, and the mean GC contents of 40.1%, 47.6%, and 47.5%, respectively (Table 1). A total of 1,925, 1,839, and 1,699 coding DNA sequences (CDSs) were annotated by the DFAST server (https://dfast.ddbj.nig.ac.jp/) with the customized parameter of DB type = *H. pylori*, respectively (Table 1).

**TABLE 1:** NHPH type stains analyzed in this study

Species	*H. suis*	*H. heilmannii*	*H. ailurogastricus*
Strain	HS1^T^ (=DSM 19735^T^)	ASB1^T^ (=DSM 24751^T^)	ASB7^T^ (=DSM 100489^T^)
Total sequence (sequencing depth)	1,689,191 bp (40×)	1,660,951 bp (95×)	1,609,065 bp (103×)
GC content	40.1%	47.6%	47.5%
CDS	1,925	1,839	1,699
Completeness	98.5%	98.48%	98.46%
Contamination	0.18%	0%	0.36%
Replicon (accession no.)	1.7 Mb chromosome (AP026769)	1.6 Mb chromosome (AP026684)	1.6 Mb chromosome (AP026687)
	43.4 kb plasmid pHS_1 (AP026770)	21.5 kb plasmid pASB1_1 (AP026685)	12.9 kb plasmid pASB7_1 (AP026688)
	43.2 kb plasmid pHS_2 (AP026771)	17.4 kb plasmid pASB1_2 (AP026686)	12.0 kb plasmid pASB7_2 (AP026689)
			3.2 kb plasmid pASB7_3 (AP026690)

The complete genome sequences of gastric NHPH species in this study should be useful in analyzing the virulence of their species as well as their strains in the future.

## Data Availability

Complete genome sequences, including chromosome and plasmid sequences, of *H. suis* HS1^T^, *H. heilmannii* ASB1^T^, and *H. ailurogastricus* ASB7^T^ have been deposited in DDBJ/ENA/GenBank under the accession nos. AP026769 (chromosome), AP026770 (pHS_1), and AP026771 (pHS_2) for HS1^T^, AP026684 (chromosome), AP026685 (pASB1_1), and AP026686 (pASB1_2) for ASB1^T^, and AP026687 (chromosome), AP026688 (pASB7_1), AP026689 (pASB7_2), and AP026690 (pASB7_3) for ASB7^T^, respectively. The raw sequence data of Illumina and ONT are available in the Sequence Read Archive under the accession nos. DRR403231 (Illumina) and DRR403234 (ONT) for HS1^T^, DRR403232 (Illumina) and DRR403235 (ONT) for ASB1^T^, DRR403233 and (Illumina) and DRR403236 (ONT) for ASB7^T^, respectively.
